# Deciphering death: a commentary on Gompertz (1825) ‘On the nature of the function expressive of the law of human mortality, and on a new mode of determining the value of life contingencies’

**DOI:** 10.1098/rstb.2014.0379

**Published:** 2015-04-19

**Authors:** Thomas B. L. Kirkwood

**Affiliations:** Newcastle University Institute for Ageing, Campus for Ageing and Vitality, Newcastle upon Tyne NE4 5PL, UK

**Keywords:** Gompertz law, mortality, ageing, demography, actuarial science

## Abstract

In 1825, the actuary Benjamin Gompertz read a paper, ‘On the nature of the function expressive of the law of human mortality, and on a new mode of determining the value of life contingencies’, to the Royal Society in which he showed that over much of the adult human lifespan, age-specific mortality rates increased in an exponential manner. Gompertz's work played an important role in shaping the emerging statistical science that underpins the pricing of life insurance and annuities. Latterly, as the subject of ageing itself became the focus of scientific study, the Gompertz model provided a powerful stimulus to examine the patterns of death across the life course not only in humans but also in a wide range of other organisms. The idea that the Gompertz model might constitute a fundamental ‘law of mortality’ has given way to the recognition that other patterns exist, not only across the species range but also in advanced old age. Nevertheless, Gompertz's way of representing the function expressive of the pattern of much of adult mortality retains considerable relevance for studying the factors that influence the intrinsic biology of ageing. This commentary was written to celebrate the 350th anniversary of the journal *Philosophical Transactions of the Royal Society*.

## 1. Introduction

At a meeting of the Royal Society of London on 16 June 1825, a paper was read by Benjamin Gompertz FRS entitled ‘On the nature of the function expressive of the law of human mortality, and on a new mode of determining the value of life contingencies', which was subsequently published in *Philosophical Transactions* [1]. In this work, Gompertz, a practising actuary, was chiefly concerned with improving the calculation of survival probabilities through the course of a statistical human life, with the aim of making more reliable the science of calculating the appropriate rates for selling and purchasing annuities [2]. Many others were also then concerned with the construction and application of life tables for actuarial purposes, but the elegance of Gompertz's analysis was the reduction of the mass of data to a strikingly simple form, henceforth known as the Gompertz equation1.1
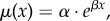
where *μ*(*x*) denotes the mortality rate at age *x*, and *α* and *β* are constants. In essence, the equation represents a force of mortality that increases progressively with age in such a manner that log *μ*(*x*) grows linearly. The term *β* might be said to describe the ‘actuarial ageing rate’, in that its magnitude determines how fast the rate of dying will increase with the addition of extra years.

Actuaries and demographers would soon incorporate Gompertz's methods into their ways of working. However, another branch of science, that of the biology of ageing, barely existed in the early nineteenth century, and it has only been within the last half-century that the Gompertz equation has become a prominent feature in the wider scientific study of patterns of life and death (box 1).

Box 1. **The life of Benjamin Gompertz (1779–1865).**

Benjamin Gompertz was born in the City of London [2]. His father was a successful diamond merchant, and his mother was Dutch by birth. As a Jew, he was excluded from the universities and privately educated. In 1797, Gompertz joined the Spitalfields Mathematical Society, later becoming its president. From 1798, he contributed regularly to the Gentleman's Mathematical Companion, winning the prize competition of that journal every year between 1812 and 1822. Gompertz was elected a Fellow of the Royal Society in 1819, served on the Royal Society's council in 1825 and 1831, and published four major papers in the *Philosophical Transactions* in 1806, 1820, 1825 and 1862.

In addition to the actuarial work for which he is best remembered today, Gompertz was an early member of the Astronomical Society of London and began in 1822, with Francis Baily, the calculation of tables of the mean places of the fixed stars. The publication of Bessel's *Fundamenta Astronomiae* anticipated them, but their work was of great importance to the construction of the Royal Astronomical Society's complete catalogue of stars.

In 1810, Gompertz married Abigail Montefiore (1790–1871), sister of Sir Moses Montefiore. They had a son, Joseph (1814–1824), and two daughters, Justina Lydia (1811–1883) and Juliana (1815–1873). In 1809 or 1810, Gompertz entered the stock exchange, leaving in 1824. When the Guardian Insurance Office was established in 1821, Gompertz applied unsuccessfully for the position of actuary, his rejection being reputedly on the grounds of his religion. In 1824, his brother-in-law, Sir Moses Montefiore, and Nathan Rothschild set up the Alliance British and Foreign Life Assurance Company, to which Gompertz was appointed actuary. He was also chief manager of the related Alliance Marine Insurance.

Gompertz's commercial activities flourished but his lasting fame derived from his philosophical interest in life tables. While others treated these only as working tools, Gompertz tried to understand the laws which produced consistent age patterns of death. His expertise in this area led to him being consulted by government, including giving evidence to the select parliamentary committees on friendly societies in 1825 and 1827, and he did important computational work for the army medical board.

After retiring from active work in 1848, Gompertz devoted much time to mathematics and science. His *Hints on Porisms* was privately published in 1850 as a sequel to earlier papers on imaginary quantities. He investigated comets and meteors, but this work was not published. He was a founding member of the Statistical Society of London in 1834 and contributed a work on human mortality to the International Statistical Congress in 1860. He was also, in 1865, one of the original members of the London Mathematical Society, for which he was preparing a paper at the time of his death.

Although appreciation today of Gompertz's work focuses on his demonstration of the exponential form of the increasing mortality rate, his 1825 publication also contained interesting observations on the pattern of mortality at very high ages and on the nature of ageing itself. Observing that the human life tables showed that from the age of 92 the annual rate of mortality was almost constant at 0.25, Gompertz reasoned that this ‘would indeed make it appear that there was no positive limit to a person's age’ and that ‘the limit to the possible duration of life is a subject not likely ever to be determined, even should it exist’ (p. 516). Furthermore, he remarked that ‘the value of life annuities of all ages above 92 would be of the same value’, for ‘if the ratio of the number of persons living from one year to the other be constantly the same, the chance of a person of any proposed age living to a given number of years would be the same, whatever that age might be’. Thus, it may be seen that Gompertz was already well aware of the phenomenon of a ‘mortality plateau’ in late life, which in recent decades has been advanced as a challenge to the ‘Gompertzian’ model.

With regard to the nature of age-related mortality, Gompertz wrote: ‘It is possible that death may be the consequence of two generally co-existing causes; the one, chance, without previous disposition to death or deterioration; the other, a deterioration, or an increased inability to withstand destruction’ (p. 517). This distinction anticipates a modification to equation (1.1), generally attributed to Makeham ([3,4]; see also [5]), which takes the form1.2
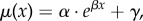
where *γ* is a constant representing age-independent mortality. It has become common within the literature on biodemography and ageing to refer to *γ* as ‘extrinsic’ mortality imposed by the hazards of the environment without regard to biological age, whereas the Gompertz term refers to ‘intrinsic’ mortality.

The object of this paper is to re-examine the legacy of Gompertz's publication [1] in relation to a number of current issues in the biology of ageing. A valuable earlier work, on somewhat similar lines, was by Olshansky & Carnes [6]. For treatises providing detailed discussion of Gompertz's work in the context of mathematical demography, see Smith & Keyfitz [7] and Impagliazzo [8] (box 2).

Box 2. **Overview of Gompertz's publication in the *Philosophical Transactions*.**

Gompertz's landmark paper [1] occupied 73 printed pages, of which 32 were taken up completely with dense numerical Tables and two with a list of Errata in his earlier publication of 1820. The 1825 publication begins by stating that it is a continuation of the previous work ‘in which I observed the near agreement with a geometrical series for a short period of time, which must pervade the series which expresses the number living at ages in arithmetical progression, proceeding by small intervals of time, whatever the law may be, provided the intervals be not greater than certain limits'. Gompertz continued: ‘I now call the reader's attention to a law observable in the tables of mortality, for equal intervals of long periods'.

The text of the work was divided into Chapters I and II, which, in keeping with contemporary style, were further subdivided into 12 and 9 ‘Articles', respectively. Each Article comprised a form of sub-section or proposition. The work as a whole contained numerous worked examples to substantiate the main points. The data were drawn from actuarial life tables from Carlisle and Northampton and reference was made to the experience of the Equitable Society, an important provider of life insurance and annuities at the time. The final page of the paper (before the concluding list of Errata) presented an Example ‘To find the annual premium to assure a life, at age a years, for 10 years, according to the Carlisle mortality, and three per cent interest’. The annual premiums for an assurance of £100 were shown to be £1 0s 4d at age 30, £1 7s 7d at age 40, £1 15s 1d at age 50, £3 1s 3d at age 60 and £7 16s 9d at age 70; these sums were expressed in British pounds, shillings (20 to 1 pound) and pence (12 to 1 shilling).

The modern formulation of Gompertz's law (equation (1.1) in this text) as *μ*(*x*) = *α* · e*^*β*x^* is not given in his 1825 paper. Instead, he presented his law as follows: ‘the numbers of persons living at the age of *x* = *_d_* · *_g_q^x^*’, where *d*, *g* and *q* are parameters, with *g*, as Gompertz emphasized, raised to the power *q^x^*. The earliest expression of Gompertz's law in the form now commonly used appears to be by Greenwood [9].

## 2. Comparative studies

The building of a connection between the Gompertz equation and the biology of ageing owes much to the work of biophysicist George Sacher [10] of the Argonne National Laboratory, whose introduction to ageing stemmed from the growing recognition during the 1950s that irradiation would shorten length of life [11]. The same recognition led physicist Leo Szilard [12] to propose the somatic mutation theory of ageing and prompted a range of studies on the effects of radiation on ageing both in animal models such as *Drosophila* (e.g. [13]) and also in human survivors of atomic bomb irradiation [14].

**Figure 1.**
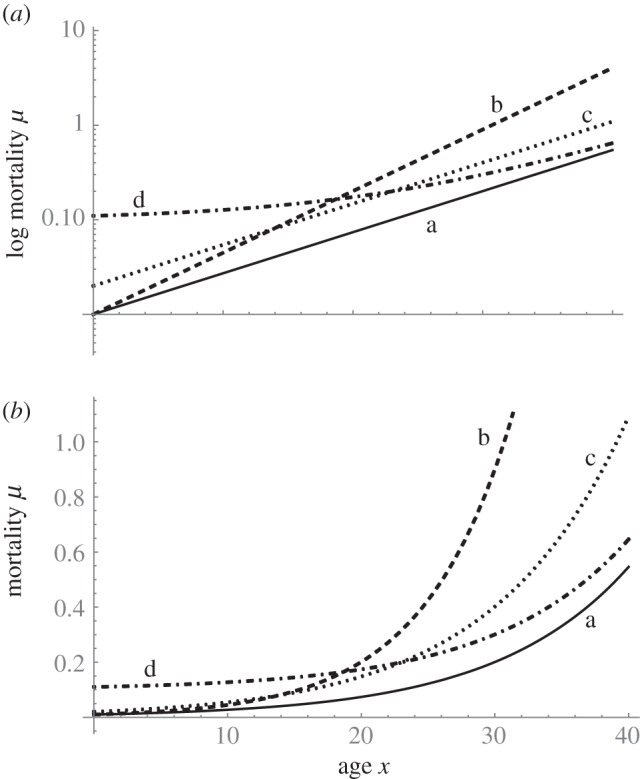
Examples of the relationship between (*a*) log mortality and age (*a*) and (*b*) mortality against age according to the Gompertz–Makeham equation (equation (1.2)). The four cases shown have parameter values as follows: a: *α* = 0.01, *β* = 0.1, *γ* = 0; b: *α* = 0.01, *β* = 0.15, *γ* = 0; c: *α* = 0.02, *β* = 0.1, *γ* = 0; d: *α* = 0.01, *β* = 0.1, *γ* = 0.1.

Sacher [10] used the Gompertz model to compare the patterns of increase in age-specific mortality rates across different species. By plotting age-specific mortality on a logarithmic scale against age (figure 1), he showed that a linear increase was generally observed, in accordance with the logarithmic version of equation (1.1), i.e.2.1
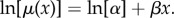

Sacher [10] termed the quantity ln[2]/*β* the mortality rate ‘doubling time, i.e. the time required for the death rate to increase by a factor of two’. The intercept ln[*α*] was termed the ‘initial death rate’, although he recognized that this extrapolation of the line back to age zero would underestimate true neonatal mortality, which would be determined by the particular causes of death characteristic of newborns. Thus, the intercept ln[*α*] was described as a ‘vulnerability parameter, for it measures the initial vulnerability to disease, before the onset of ageing’, and does not relate to early-life mortality itself, which varies greatly with the physiology, morphology and reproductive biology of different species. Indeed, Gompertz himself recognized that equation (1.1) served only to describe adult mortality.

By comparing survival data from a range of mammalian species, Sacher [10] reported that the mortality doubling time was 220 days for the house mouse (*Mus musculus*) but 3100 days for humans (US white females, 1969). Such analyses have become a generally accepted and useful tool for comparing different species with regard to their rates of ageing [15,16], especially among those species that exhibit patterns of biological senescence that are broadly familiar from decades of studies in vertebrates and other laboratory models. However, it was early recognized that not all species conformed to the Gompertz model, especially those where growth patterns are indeterminate and where there may be either an absence of apparent senescence (e.g. in *Hydra* [17]) or even a decline in mortality rates with increasing age, a possibility first seriously considered by Vaupel *et al.* [18]. A recent compilation of biodemographic data across a very wide range of organisms confirms the diversity of mortality patterns [19]. Nevertheless, the application of the Gompertz model as a yardstick continues to serve as a means of determining whether or not ageing, as it has been conventionally recognized, is a feature of a species' life history.

**Figure 2.**
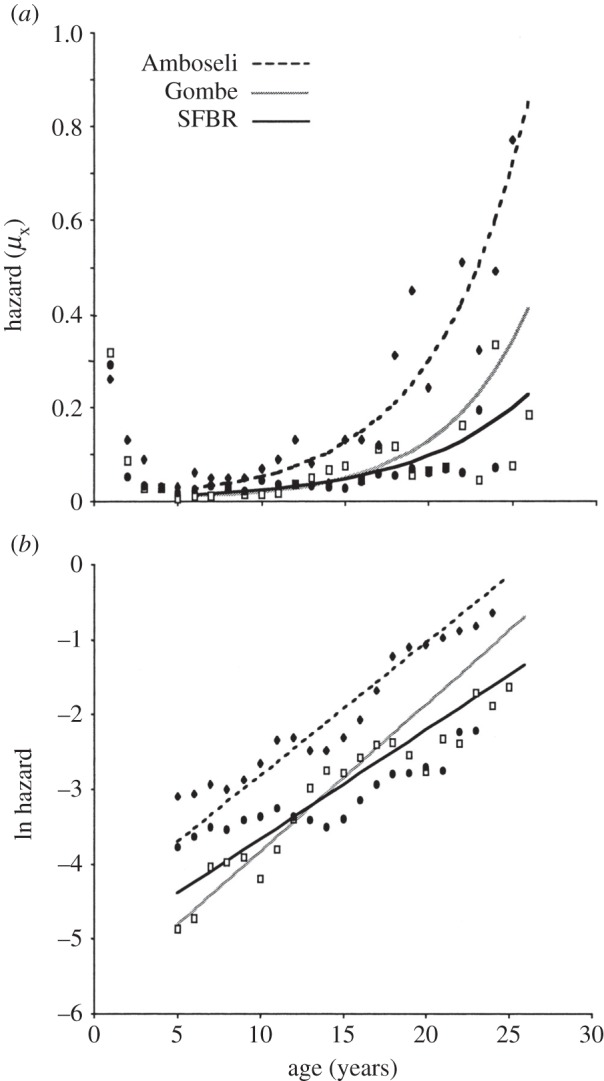
An example of a comparison of age-related mortality patterns in three populations of baboons (*Papio hamadryas*), based on one captive (Southwest Foundation for Biomedical Research; SFBR) and two free-living populations (Amboseli National Park, Kenya, and Gombe National Park, Tanzania). Adapted from [20] © the National Academy of Sciences.

Comparative use of the Gompertz model has also been useful when analysing factors that may alter the mortality patterns within a species (figure 2). Sacher [10] examined mortality curves for groups of mice exposed to various levels of exposure to nuclear radiation. The data, when plotted with mortality on a logarithmic scale, showed a series of parallel lines. This was consistent with the interpretation that the radiation did not alter the underlying rate of actuarial ageing but added a quantum of damage that was equivalent to altering the basal vulnerability of the animal. With regard to the nature of the underlying damage, Sacher [10] discussed extensively how the effects on the Gompertz model could be related to the molecular mechanisms at play, especially with regard both to lesions introduced by radiation and also to their relationship to cancers arising in the various organs of the body. He was well aware of the pitfalls of overly simplistic identification of radiation damage and ageing: ‘Exposure to ionizing radiation produces a kind of primary injury that remains permanently imbedded in the molecular constitution of the organism. This fact made it possible to find the mathematical transformation that makes radiation injury additive with itself and with the injury of natural ageing, and this led, in turn, to the development of the theory of the probabilistic relationship between the level of cellular–molecular injury in a population and its expected death rate. These conclusions are quite general and can be carried over to the analysis of life prolongation by anti-ageing therapies. However, present evidence indicates that ageing is not due to the same molecular lesion as radiation life shortening. Thus, the efficacy of a drug as a protective agent against radiation life shortening is not necessarily related to its efficacy in life prologation’.

More recently, the Gompertz model has been used to distinguish the ways in which altering temperature and varying nutrient levels affect the trajectories of age-specific mortality in *Drosophila* [21,22]. Both treatments affected lifespan; however, whereas temperature affected the slope *β* of the log–mortality relationship with age, altering the diet resulted only in a shift in intercept. Furthermore, by switching flies between temperatures and diets, Mair *et al.* [21] found that those switched between temperatures permanently bore the mark of their thermal history, while those switched between diets rapidly and completely adopted the mortality trajectory of flies chronically maintained on the new diet. These results, as was later demonstrated through analysis of various molecular protein and other biomarkers [22], point to opportunities to relate the effects of interventions that alter ageing profiles to the nature of the underlying molecular damage that is involved.

Analysis using the Gompertz model has also been applied to examining the trends in human longevity. Jones [23] compared mortality patterns from India in 1900, Mexico in 1940, Sweden in 1949, and the USA in 1900, 1940 and 1950. Despite large differences between these populations in the levels of mortality in earlier life, the slopes in a graph of log mortality rate against age showed a close approximation to parallelism after mid-life (40 years), with a mortality doubling time consistently around 8 years. Even more strikingly, comparison between mortality profiles of Australian prisoners in Japanese prisoner of war camps in 1945, where conditions were notoriously harsh, showed parallel increases with age, when plotted as logarithms, with the mortality profile of civilians in Australia at the same time. In other words, despite a substantial increase in mortality rate across the age span, the underlying rate of actuarial ageing appeared to be the same in both populations [24]. Vaupel [25] provided evidence that the slope parameter *β* in equation (2.1) is the same for all human populations today and in the past, whereas it is *α* that varies. More recently, there has however been growing recognition that interactions exist between conditions experienced in early life and later outcomes in terms of age-related disease. Analysis by Beltrán-Sánchez *et al.* [26] of historical human cohorts suggested the existence of a negative correlation between early cohort mortality and *β*.

## 3. A law of nature?

The neatness with which the Gompertz equation described age-related mortality, and its simple formulation with just two parameters, prompted speculation that it might somehow capture a fundamental law of nature, akin to some of the laws of physics. The early twentieth century witnessed numerous attempts to discover the nature of this law, epitomized by Pearl [27,28] (see review by Olshansky & Carnes [6]). Although these early efforts ultimately failed, the search for an explanation of the Gompertzian pattern has continued and attempts are still made to justify the existence of ‘Gompertz's survivorship law as an intrinsic principle of aging’ [29].

Some of the most interesting attempts to explain the existence of Gompertzian mortality start from the engineering perspective of ‘reliability theory’, which enables the age-related failure kinetics of an object to be understood through analysis of its construction (reliability architecture) and the reliability (or expected time to failure) of its component parts. If the continued viability of the object depends on the operation of each and every one of its components, such that the first failure is lethal, then mortality will be a simple matter of essentially exponential decay. This is akin to the hazard represented by extrinsic accidents, which kill without warning. If, however, the object is composed of collections of irreplaceable but redundant components, such that the failure of one does not automatically cause death, then the object can accumulate a growing number of non-lethal component failures. Over time, this will result in an increasing probability that the last of an initially redundant cluster will fail, bringing about death. There are of course many different scenarios that might be considered, where the efficiency of a cluster becomes progressively more compromised as its components fail one by one, or where components operate in networks, or where repair and/or renewal is possible.

Formal analysis of an idealized model of highly redundant systems with multiple defects was undertaken by Gavrilov & Gavrilova [30], who showed that reliability theory could explain why mortality rates of biological organisms increase exponentially with age according to the Gompertz equation. In the abstract case considered by Gavrilov & Gavrilova [30], the organism is represented as a collection of a certain number of irreplaceable ‘blocks', each block comprising a certain number of mutually substitutable ‘elements' so that failure of a block occurs only when all elements of the block have failed. Whenever one of the blocks fails, the organism dies. With this kind of model, it could be shown that the failure rate of an organism initially grows exponentially following the Gompertz model. Further elaboration extended the analysis to cases where the system was from the beginning compromised by a number of pre-existing faults, consistent with the fact that some inherited gene defects (mutations) will be transmitted to the progeny. In general, reliability models predict that the mortality pattern of the organism should conform at least initially to the Gompertz model, although an alternative model—the Weibull [31] power law model—might also be encountered, especially in cases where organisms are assumed to be relatively free of initial flaws and defects [30].

Variants of the reliability-theory approach have further developed idealized models for the accumulation of damage within biological networks, notably the ‘nested binomial model’ of Milne [32], which assumes the organism to comprise a set of functions connected ‘in series' such that the failure of any single function results in death. The probability that a function fails at any time is assumed to depend upon the interaction of the external risk environment and its intrinsic vulnerability, with each functional unit being to some degree internally redundant—that is to say it has, in a healthy and vigorous state, a capacity that exceeds most normal demands. As Milne showed, these assumptions can cover a very wide range of biological cases and the model has considerable, broad-brush explanatory power. In particular, Milne [32] suggested that this approach could explain not only why mortality rates increase exponentially for so much of life in so many species, and why accelerating mortality can be reconciled with linear rates of loss of physiological function, but also why Gompertzian mortality doubling times appear to become shorter when levels of absolute mortality fall, a phenomenon originally described by Strehler & Mildvan [33].

While reliability-theory models treat the organism in an idealized abstraction, molecular and cellular studies are gradually throwing light on the kinds of defects that contribute to the cumulative erosion of functionality that occurs during the ageing process. For many years, these data have grown in a patchwork way, through the pursuit of specific single-aspect theories of ageing, such as the somatic mutation theory and free-radical mitochondrial theory. Latterly, however, it has become clear that single-aspect theories have limited explanatory power and that, instead, there is a need to develop network theories in which the individual mechanisms interact with each other, using the methods of systems biology [34–36]. Growing evidence from combined experimental and modelling studies confirms the value of this approach (e.g. [37,38]), so that eventually it may become possible to connect Gompertzian mortality patterns with the underlying primary mechanisms.

## 4. Limits of survival and late-life mortality plateaux

The original derivation of Gompertz's model was based on the analysis of life tables comprising death records for large numbers of individuals. However, as noted earlier, Gompertz was also interested in the question of whether an upper limit to human lifespan exists and in the shape of the mortality pattern at very high ages when the number surviving is much smaller. These questions have received considerable attention recently as human longevity continues to increase [39–41] and following the observation that not only in humans but also in invertebrates there is a plateau of static (or even declining) mortality rates at the very highest ages [42,43].

The popular notion that there might be some in-built limit to human survival does not stand up well to the generally accepted logic that argues against the idea of any actively programmed control of the ageing process [44]; although there is no doubt that genes influence longevity, they appear to do so not through a strict ‘clock’ but through regulating maintenance and repair functions, particularly through metabolic pathways that influence these processes. Thus, the distribution of survival at the highest ages most likely reflects the variation inherent in statistical extreme-value distributions [40].

Thatcher [40] examined data accumulated at advanced human ages within the Archive on Population Data on Aging [45]. That the mortality pattern follows the Gompertz model closely during the major portion of the adult lifespan reflects the fact that such dynamics are a consistent prediction of multiple models, including those derived from reliability theory. At the extremes, however, other models appear to fit as well or better. The models that were compared were: the Gompertz model; the three-parameter logistic model *μ*(*x*) = *z*/(1 + *z*) + *γ*, where *z* = *α ·* exp(*βx*); the Weibull [31] model *μ*(*x*) = *α* *· x*^*β*^; and a model by Heligman & Pollard [46] which for high ages can be written in the form logit(*q*(*x*)) = *α* + *βx*, where *q*(*x*) in standard actuarial notation is the probability of dying within 12 months of reaching age *x*. It should be noted that although the same Greek letters are used in these varying models, they represent different parameters within each model. Furthermore, unlike the nested binomial model of Milne [32], described earlier, the models considered by Thatcher [40] lack assumptions about underlying biological mechanisms and were chosen simply for their capacity to fit the observed data. Thatcher [40] concluded that the closest description of the observed pattern of mortality at the highest attained ages was seen with the three-parameter logistic model.

Whichever model best fits the data for survival to extreme human ages, the phenomenon of the apparent plateau in late-life mortality requires explanation. One attractive solution to this enigma is population heterogeneity [39,47,48]. If the population comprises a set of individuals who differ in the inherent robustness or rate of ageing, the frailest individuals tend to die soonest, leaving the most robust individuals to predominate at the oldest ages. In such a scenario, it is possible for a late-life mortality plateau, or even a decline, to be seen at the population level, even if the various individuals that comprise the population all experience an increasing probability of dying with advancing age.

Various attempts have been made to test the contribution of population heterogeneity to mortality deceleration at late ages, especially in invertebrates. Results to date are mixed. Khazaeli *et al.* [49] performed an experiment with *Drosophila* where the population was fractionated to reduce as much as possible the degree of pre-adult environmentally induced heterogeneity among individuals of a genetically identical cohort. From a total of 106 fractionated and control populations, consisting of more than 50 000 individuals, 101 populations exhibited a significant amount of mortality deceleration late in life. These results suggested that heterogeneity accrued during early development was not a major factor contributing to mortality deceleration in later life. By contrast, in the nematode *Caenorhabditis elegans*, techniques have been developed to visualize ‘hidden heterogeneity’ that may accrue during the lifespan within isogenic populations [50]. Such an accumulation through life of somatically acquired heterogeneity is inherently predicted through the action of ‘chance’ developmental variations and age-related molecular damage [51,52]; furthermore, reporter constructs designed to reveal molecular stress responses within individual worms in isogenic populations indicate that such responses are predictive of lifespan, consistent with the emergence of functionally significant underlying heterogeneity that impacts on survivorship [53]. Further support for the role of heterogeneity in causing late-life mortality deceleration in nematodes comes from experiments by Chen *et al.* [54], which manipulated sources and rates of externally imposed mortality.

## 5. Benjamin Gompertz: a concluding appreciation

It is rare for a single publication to provide the foundation for one field of research and even rarer for it to underpin two. Indeed, following the development by Winsor [55] of Gompertz's equation as a basis of growth curves, both for biological and economic phenomena, the same publication has had an influence of even broader scope (box 3).

Box 3. **The breadth of Gompertz's scientific legacy.**

A search using the search term ‘Gompertz’ of the databases of scientific literature, such as PubMed and Web of Science, reveals the diversity of scientific studies that continue to draw upon the ideas contained within Gompertz's 1825 publication. Additionally, his influence pervades the specialized literature and working methods of actuaries.

The primary focus of this commentary has been on the significance of Gompertz's work for studying patterns of mortality. However, as will be seen immediately from literature searches, a very large number of studies make use of Gompertz's work in the form of growth curves [55]. The following is a small selection of the titles of publications retrieved from a search of PubMed:

— Modeling senescence changes of the pubic symphysis in historic Italian populations: A comparison of the Rostock and forensic approaches to aging using transition analysis.
— Inactivation and sublethal injury kinetics of *Staphylococcus aureus* in broth at low temperature storage.
— The distribution of incubation and relapse times in experimental human infections with the malaria parasite *Plasmodium vivax*.
— Environment-dependent microevolution in a Mediterranean pine (*Pinus pinaster* Aiton).
— Mathematical modeling of tumor growth and metastatic spreading: validation in tumor-bearing mice.
— Age trajectories of mortality from all diseases in the six most populated countries of the South America during the last decades.
— Mortality risk and survival in the aftermath of the medieval Black Death.
— Predicting mortality in people with Type 2 diabetes mellitus after major complications: a study using Swedish National Diabetes Register data.
— Genetic parameter estimates of growth curve and reproduction traits in Japanese quail.
— Parametric likelihood inference for interval censored competing risks data.
— Use of the modified Gompertz equation to assess the *Stevia rebaudiana* Bertoni antilisterial kinetics.
— Growth patterns of Italian local chicken populations.

Although Gompertz's paper was not alone in forming the basis for actuarial science, it has played a pivotal role in doing so and is very well known to practising actuaries today. Within the biology of ageing, Gompertz's work has had an even more distinctive impact, notably through the early recognition of its significance by George Sacher. The subsequent field of the biodemography of ageing has grown largely through exploring its implications and through ‘proving the rule’ by exploring cases where it does not so well describe the patterns of mortality. As one leading proponent put it ‘The more the Gompertz equation is studied, the more interesting it becomes' (J. W. Vaupel 2014, personal communication). Today, as actuaries and biogerontologists converge in addressing the challenges of understanding the continual increase in human longevity and the underpinning science of ageing, the twin legacies of Benjamin Gompertz are beginning to converge. It is about time.
